# Beyond boundaries: a location-based toolkit for quantifying group dynamics in diverse contexts

**DOI:** 10.1186/s41235-025-00617-6

**Published:** 2025-02-21

**Authors:** Seth Elkin-Frankston, James McIntyre, Tad T. Brunyé, Aaron L. Gardony, Clifford L. Hancock, Meghan P. O’Donovan, Victoria G. Bode, Eric L. Miller

**Affiliations:** 1 Cognitive Science Branch, US Army DEVCOM Soldier Center, Natick, MA USA; 2 Biomechanics and Engineering Branch, US Army DEVCOM Soldier Center, Natick, MA USA; 3 Center for Applied Brain and Cognitive Sciences, Tufts University, Medford, MA USA; 4 Department of Electrical and Computer Engineering, Tufts University, Medford, MA USA

## Abstract

Existing toolkits for analyzing movement dynamics in animal ecology primarily focus on individual or group behavior in habitats without predefined boundaries, while methods for studying human activity often cater to bounded environments, such as team sports played on defined fields. This leaves a gap in tools for modeling and analyzing human group dynamics in large-scale, unbounded, or semi-constrained environments. Examples of such contexts include tourist groups, cycling teams, search and rescue teams, and military units. To address this issue, we survey existing methods and metrics for characterizing individual and collective movement in humans and animals. Using a rich GPS dataset from groups of military personnel engaged in a foot march, we develop a comprehensive, general-purpose toolkit for quantifying group dynamics using location-based metrics during goal-directed movement in open environments. This toolkit includes a repository of Python functions for extracting and analyzing movement data, integrating cognitive factors such as decision-making, situational awareness, and group coordination. By extending location-based analytics to non-traditional domains, this toolkit enhances the understanding of collective movement, group behavior, and emergent properties shaped by cognitive processes. To demonstrate its practical utility, we present a use case utilizing metrics derived from the foot march data to predict group performance during a subsequent strategic and tactical exercise, highlighting the influence of cognitive and decision-making behaviors on team effectiveness.

## Introduction

Location-based metrics provide rich insights into individual and group-level behavior across diverse contexts, environments, and activities. Their value, coupled with the widespread availability of cost-effective Global Positioning System (GPS) technology, has led to their widespread adoption across a range of interdisciplinary applications in fields ranging from sports analytics (Low et al., 2020; Torres-Ronda et al., 2022) to animal ecology (Cushman & Huettmann, 2010; Nathan et al., 2008; Seidel et al., 2018).

However, despite the proliferation of positional tracking technologies and advanced mathematical modeling methods, analyzing the behavioral dynamics of groups of humans working toward a common goal as they move through an unbounded environment remains a challenge. Here, we define collective goal-seeking as human actions that aim to achieve personal, social, or occupational objectives. Many cognitive processes underpin this behavior, including planning, decision-making, and intentionality, distinguishing it from instinctual behaviors observed in animal populations, such as food-seeking, migration, or mating. Similarly, we use the terms “unbounded” or “unconstrained” to refer to a region of space where there is no clearly defined boundary. In this paper, we focus on groups of humans working collectively toward a shared objective or goal over a non-specified area. This includes how individuals within a group coordinate behavior in response to dynamic environments, encompassing perception and attention, effective communication, decision-making, adaptability to new information or obstacles, and role differentiation, such as leaders and followers.

For example, during military operations, Soldiers coordinate and direct their actions to monitor their surroundings, avoid and react to threats, or occupy a strategic position. In search and rescue contexts, first responders coordinate and direct their actions to establish containment points, search zones, and update search plans as new information is discovered. Prominent theories of group cognition, such as Shared Mental Models (SMMs) (Cannon-Bowers et al., 1993) and Transactive Memory Systems (TMSs) (Hollingshead, 1998), provide valuable frameworks for understanding how teams share knowledge, align goals, and distribute cognitive responsibilities. These frameworks explain the mechanisms that enable teams to perform effectively, emphasizing how individual capabilities contribute to collective understanding, shared expertise (Gevers et al., 2020) and effective leadership (Toader & Martin, 2023).

A more contemporary framework, Interactive Team Cognition (ITC), suggests that team cognition emerges from interactions between team members rather than as an aggregation of individual knowledge and contributions (Cooke et al., 2013). This dynamic view of team cognition has spurred the need for real-time assessments of team interactions (Cooke et al., 2023). For example, Gorman et al. (2020) developed a technique to measure real-time team cognition by analyzing team communication, successfully quantifying individual-level contributions to team adaptation (Gorman et al., 2020). However, there remains a critical need for approaches that can unobtrusively and effectively measure team interactions, particularly in operational contexts.

In military scenarios, such approaches must capture the dynamic interplay between individual and group behaviors, especially in unbounded or semi-constrained environments. Novel methods, such as location-based metrics and the use of spatiotemporal features, hold potential to enhance our understanding of cognitive aspects of group interactions, including training, operational planning, and the development of predictive tools to optimize collective performance (Bloch et al., 2023; Bustos et al., 2021; Giles et al., 2023).

To bridge this gap, we first survey current interdisciplinary methods and metrics utilized in sports analytics, ecology, and human mobility analytics. We then identify techniques that can be directly applied or serve as foundation for innovative approaches, with the goal of creating a comprehensive toolkit to understand and characterize group dynamics using location-based metrics in naturalistic environments during goal-directed activities. Next, we describe a set of functions for extracting and analyzing movement data, including velocity-based, spatiotemporal, clustering, directional, and movement regulatory features. Finally, as a motivating example and our primary use case, we apply our novel methods to the military domain. Specifically, we focus on the scenario of a small groups of Soldiers conducting a foot march, often referred to as a “ruck” march, as part of a larger US Army research and training exercise.

### Approaches in movement ecology

In ecology, researchers have used tracking technologies (such as radio telemetry and GPS) to monitor human–wildlife interactions, identify and preserve habitats, and observe disease spread over relatively unconstrained areas (Seidel et al., 2018). Many metrics and methods developed from these foundational ecological applications can translate to understanding individual and group-level human dynamics. First, methods derived from path-level movement analysis, such as step-length or turning angle, can serve to characterize how humans move through unconstrained environments (Calenge et al., 2009). Beyond primary characterization, secondary statistics, such as net squared displacement (NSD) (Bunnefeld et al., 2011), can identify behavioral or functional collective states, such as migratory vs. sedentary behavior or resting vs. foraging (Bastille-Rousseau et al., 2016). Primary and secondary measures of individual movement provide the foundation of location-based analysis and are critical for understanding group dynamics.

A set of related methods focuses on segmenting movement patterns to pinpoint shifts in behavioral states. Path segmentation techniques, such as behavioral change point analysis, often use time-series analysis to identify shifts in movement patterns that relate to state changes due to varying environmental conditions (Gurarie et al., 2009, 2016). With respect to human movement, change point detection approaches (Cheng et al., 2020) offer a means to classify changes in movement, such as rucking in a fatigued or injured state.

Another analytical approach concerns the study of group dynamics and how individuals influence collective behavior. In bird flocking research, directional correlation function analyses have been used to define hierarchically organized movement and identify leader–follower interactions (Nagy et al., 2010). This approach models how a leader’s movement influences group behavior with a predictable time delay. By incorporating cognitive aspects such as decision-making, situational awareness, and communication, similar approaches can inform human interactions within collectives to better characterize complex group dynamics and emergent organizational structure during coordinated action. Understanding the cognitive processes behind these interactions is of great utility to team sports and military applications where a hierarchical command structure is strictly enforced, as it allows for the optimization of strategies based on how individuals perceive, process, and react to information within the group.

While we focus here on GPS for tracking movement over the spatial and temporal scale, it should be noted that many other sources and types of data are leveraged in animal movement ecology. For example, ecology researchers can use accelerometry to measure activity levels and behavior transitions, Argos tags and satellite telemetry to track long-distance migration patterns (Hoenner et al., 2012), network analysis to understand the flocking and herding behavior of animal groups (Farine & Whitehead, 2015), agent-based modeling to simulate movement and develop and validate models (Tang & Bennett, 2010), and environmental deoxyribonucleic acid (DNA) to study long-term movement patterns from genetic traces of animals (Thomsen et al., 2012). Each technique carries its own set of advantages and disadvantages based on the temporal and spatial extent of tracking needs. However, for understanding human group dynamics during collective action, GPS stands out for its low cost, reliability, and ease of use.

### Approaches in sports analytics

Like in ecology, location-based metrics are a powerful tool for analyzing athlete and team performance. When focusing on individual athletes, these metrics offer insights into training and competition loads and aid in the mitigation of injury risks (Halson, 2014; Vanrenterghem et al., 2017). Similar to movement ecology, primary and secondary GPS features can be used to develop behavioral classifiers, enabling tasks such as injury forecasting (Rossi et al., 2018), tactical performance analysis (Folgado et al., 2018), training recommendations (Torres-Ronda et al., 2022), and group cognition, leadership, and team organization (Gorman et al., 2020; Toader & Martin, 2023; Zhang et al., 2023).

Of course, individual athletes are often members of teams. Thus, there is great interest in understanding how groups of athletes collectively interact and perform. Team performance metric categories include team centrality, synchrony, and player–player interactions (Folgado et al., 2014; Frencken et al., 2011; Silva et al., 2014). Measures of centrality analyze the centroid or mean lateral and longitudinal position of groups of players, such as those of offensive and defensive players of opposing soccer teams. Analyzing the movement of individual players in relationship to the group’s geometrical center provides valuable insight into individual (Silva et al., 2014) and group-level contributions to tactical behavior (Folgado et al., 2014; Frencken et al., 2011). Similarly, stretch index, or the moving average distance of individual players to the group centroid, can measure sports team dynamics at discrete time points driven by between-player interactions, such as during ball passes or communication (Folgado et al., 2014; Frencken et al., 2011). In an operational or military context, stretch index, or total area, may provide valuable information reflecting tactical readiness or vulnerability to attack. The total spread, or distance, between team members could impact response to enemy contact by influencing communication and situational awareness.

Inter-individual coordination and team dynamic metrics can also capture emergent tactical behaviors within groups of players. For example, characterizing network interactions between soccer players using approximate entropy reveals patterns of tactical behavior, thereby highlighting how individual player performance contributes to overall team effectiveness (Gonçalves et al., 2017a, 2017b). Notably, tactical behavior and team synchronization are influenced by the size of the playing field, where smaller, more restrictive playing fields are associated with reduced synchronization among individuals (Gonçalves et al., 2017a, 2017b; Gonçalves et al., 2017a, 2017b; Olthof et al., 2018). This suggests that such approaches for analyzing group dynamics may not directly translate to scenarios where the environmental bounds are unconstrained or unknown. Instead, methods borrowed from movement ecology may be more appropriate.

Lastly, complementary approaches can provide deeper understanding of athlete performance and team dynamics in sports. Biomechanical analysis tools and techniques, including motion capture, force plates, and wearable sensors, provide rich insights into athlete movements and technique, aiding in injury prevention and performance optimization (Robles-Palazón et al., 2023; Ye et al., 2020). Video analysis, coupled with computer vision algorithms, offers a comprehensive view of game events, tactics, and player interactions, enabling strategic planning and tactical adjustments (Low et al., 2020). Machine learning and predictive analytics leverage large datasets to forecast outcomes and optimize strategies, while physiological monitoring systems track athlete exertion and recovery, guiding training programs. Furthermore, measuring group cognition can also provide dynamic insights to understand performance and team dynamics beyond individual metrics. For example, assessing abilities to rapidly and effectively coordinate behavior, like communication (Gorman et al., 2020), and decision-making (Cannon-Bowers et al., 1993) in response to changing situational demands are indicators of team effectiveness. These approaches, independently and combined with GPS-based metrics, provide complementary insights into athlete and team performance, informing decision-making and ultimately driving athlete and team success. For example, location-based metrics may reflect adaptability and decision-making by quantifying how individuals adjust to group needs in dynamic environments.

### Characterizing goal-oriented movement in unconstrained space

While many methods from ecology and sports analytics are relevant at first glance, such as those used in the analysis of pack and flock movement, this prior work explicitly or implicitly incorporates bounds, such as playing field boundaries in a sports and habitats and/or encampment areas in ecology. Thus, these current methods are not equipped for spatial analysis of moving groups, especially when traveling along an unbounded path.

To address this issue, prior research has applied spatial transformations that incorporate the location and heading direction of the group’s centroid (Andrienko et al., 2013). This method is adequate if the group is moving in a straight line but may become inaccurate if there are significant turns along the path. Group members in the front or back may pass a turn in the path before or after the centroid. The further in front or behind the centroid an individual is, the more inaccurate the spatial transformation of their position will be. We extend this analytic approach by introducing a method that incorporates the trajectory of the group’s centroid into the spatial transformation. This allows for an accurate spatial transformation with respect to the group’s path, rather than the heading direction of the centroid. We term this methodology the Path-Adapted Coordinate System (PACS). By providing a heading-independent coordinate system, this novel spatial transformation enables the application of many sports analytics methods to coordinate group movement in unconstrained or semi-constrained spaces, especially when the group is spread out along a path, such as cycling teams navigating among each other.

Below we describe a toolkit of motion features that both draws on the state of the art in this field and extends it to address unique challenges arising when considering group motion in unbounded domains. To evaluate the effectiveness of the features discussed in this paper, we investigate their ability to predict group-level performance during a simulated force-on-force battle drill, a training exercise where military units engage in simulated combat in a controlled environment, based on prior group movement during the ruck march. Overall, this research contributes to the expanding field of location-based analytics to non-traditional domains, such as small unit military tactics, first responders, or robotic swarms. By quantifying movement patterns and group interactions, this study provides a robust framework for analyzing and understanding the cognitive underpinnings of collective behavior in naturalistic settings. Our toolkit, Feature Learning for Organized Collective Kinetics (FLOCK), provides a comprehensive set of movement and group behavior metrics to further understand collective movement and the impact of individual contributions to group behavior and other emergent properties. The package can be found on GitHub at https://github.com/Tufts-University/FLOCK.

## Methods

### Participants

Participants included 70 active-duty members of the US Army between the ages of 18 and 23 years (all male). Written informed consent was obtained, and the US Army Combat Capabilities Development Command Armaments Center Institutional Review Board and the Army Human Research Protections Office approved all procedures.

### Data collection

#### Ruck march

Nine groups of participants, each comprised of six to nine members, participated in a 7-mile foot march (“ruck march”) across level terrain as part of a larger outdoor research activity managed by the US Army DEVCOM Soldier Center (Natick, MA, USA). Participants carried standard-issue military gear including a load carrier vest, rucksack, and rifle, with an average weight load of 87 lbs. A single designated leader (i.e., squad leader) guided the group's movement and scheduled breaks, consistent with US Army doctrine (Department of the Army, 2022). GPS data were collected from each Soldier with a body-worn Polar Team Pro tracking system (Polar Electro Inc., Bethpage, NY). The Polar Team Pro includes GPS coordinates sampled at 10 Hz, tri-axial accelerometer, gyroscope, and magnetometer data, sampled at 200 Hz, and heart rate. Only GPS data were processed for this study.

#### Situational Training Exercise (STX)

Within 24 h of completing the ruck march, the same groups participated in a simulated force-on-force Situational Training Exercise (STX). The STX required groups to execute a “Squad Assault/Attack” battle drill (Battle Drill 2A), which involves a movement through forested terrain until engagement with an enemy force (Department of the Army, 2016). During the battle drills, groups were evaluated by four military subject matter experts (i.e., observer–controllers (OCs)). The OCs observed and rated Soldiers’ performance on all events and activities against standardized procedures and task steps, including moving toward the objective while maintaining security, establishing an assault position, initiating and advancing on the enemy, clearing the objective, consolidating and reorganizing, and reporting the mission status to higher command.

### Data preprocessing and feature extraction

#### Preprocessing

GPS data used for this study were acquired as *.GPX (GPS eXchange Format) and converted to CSV (comma separated value) with one data point per row. Each data point is from one individual and includes a time, an identification label, and geographic coordinates (latitude and longitude) or Universal Transverse Mercator (UTM) coordinates. Using UTM coordinates, we represented data as meters (m) along northing and easting axes rather than degrees of latitude and longitude to minimize distortions in distance and area calculations since the Earth is not a perfect sphere.

#### Smoothing and interpolation

GPS data have been shown to be accurate and reliable for measuring movement patterns in sports within confined areas (Duffield et al., 2010). However, during an open activity where the GPS device may experience some interference from tree canopy or buildings (i.e., urban canyons), more preprocessing is needed to smooth noisy datapoints and infer missing datapoints. The FLOCK toolkit includes functions for spline smoothing as it is the most common method for GPS smoothing across industries and can also be used for interpolating missing datapoints (Early & Sykulski, 2020; Linke et al., 2018; Ravankar et al., 2018). Moreover, data smoothing has a significant impact on feature extraction, particularly velocity-based features, and thus, different algorithms can have varying impacts on the resulting metrics. See Appendix for more details on preprocessing methods.

#### Rest detection

The features discussed in this paper are all related to group movement. To remove epochs without movement (i.e., breaks, rest periods) and thus facilitate movement-related feature extraction, we first segmented time periods when the group was in motion by identifying break periods using the stop detection feature from the MovingPandas Python package (Graser, 2019). The criteria for identifying a break or rest period may vary depending on the context of the data. If there are no breaks in the movement activity, the break detection function will split the dataset in half, allowing for feature extraction where multiple movement periods must be compared, such as consistency features. In human movement data, such as a loaded ruck march, breaks should be identified when an individual stays within a 100 m^2^ area for at least 120 s (s). This criterion suggests that breaks occur when the average speed is below 0.833 m/s, which aligns with literature on human movement. In a systematic review by (Murtagh et al., 2021), outdoor walking speed for humans was reported to be around 0.82 m/s for a very slow stroll. Therefore, parameters in the MovingPandas package were tailored to reflect these real-world data.

To identify rest periods on a group level, we first find rest periods for each individual separately. Once we have those individual rest periods, we identify overlapping segments that include all members of the group and combine them into a group rest period, where the earliest start time is the beginning of the break and the latest is the end of the break. This new group-level rest period represents the time that the first individual arrives to the break area to the time the last individual leaves the area (note that these are typically different individuals). Using the group-level break times, we extract the time in between as "movement periods." We further refine these periods by trimming 30 s from either side, ensuring that we are excluding transition periods between movement and rest states.

#### Path-Adapted Coordinate System (PACS)

Common metrics used in sports analytics have been generally developed within constrained environments and with a set "heading" direction, usually two teams facing one another (e.g., playing fields, courts). Consequently, it is necessary to first project GPS location data to a space such that the heading direction of the group remains constant to leverage these metrics. Current methods include centroid-based transformations, such setting the centroid as the origin (0,0) and using the immediate heading direction of the centroid as the group’s heading direction for each timepoint. This method works if the group is very close together or if the group is moving in a straight line. Real-life group movement scenarios rarely consist of a straight path or a tight-knit group. As we demonstrate, artifacts arise in the transformed data from this method as the centroid path encounters a turn before or after some group members reach the turn. If a group member is behind the centroid when they make a turn, that group member’s transformed location will be shifted in the opposite direction of the turn, resulting in an inaccurate quantification of their position relative to the rest of the group.

**Fig. 1:**
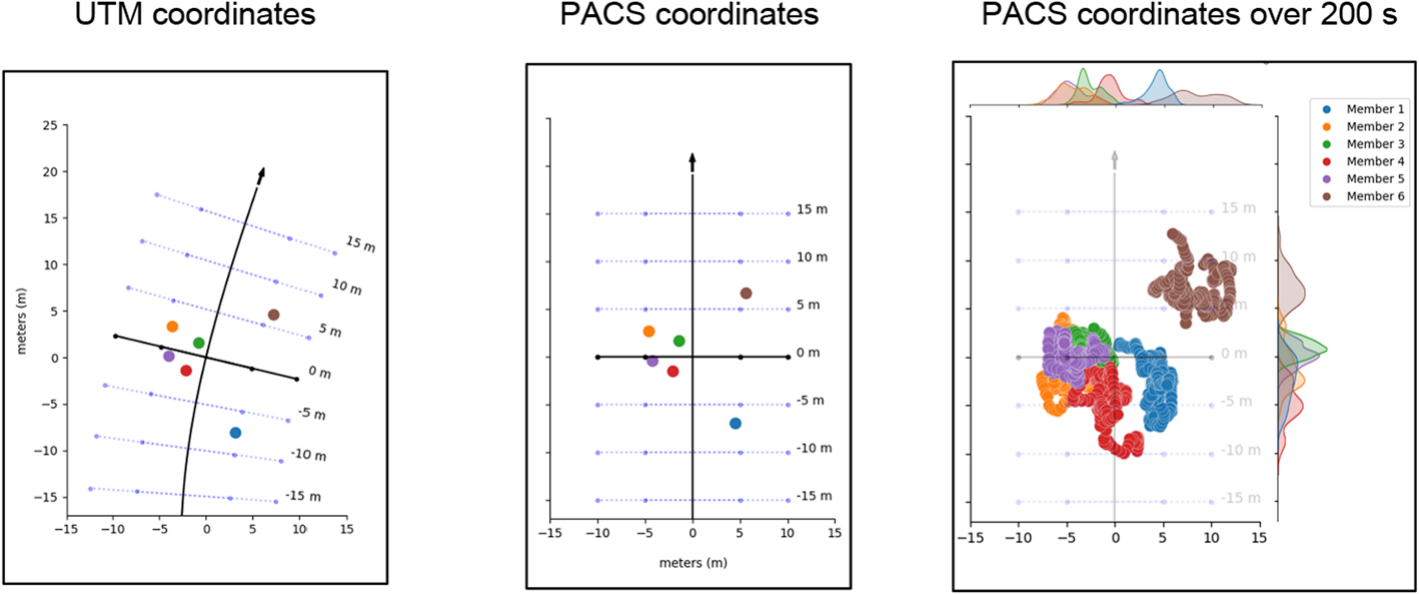
PACS visualization: The left figure (UTM Coordinates) shows raw UTM data from one group of team members at one timepoint. The line represents the smoothed path, calculated from centroid positions over time. The middle figure (PACS coordinates) shows the PACS locations of the same group of team members at the same timepoint. The right figure (PACS coordinates over 200 s) shows PACS locations for the group of team members during one movement period of 200 s, with marginal distributions plotted as histograms

To mitigate the prevalence of these transformation artifacts, we introduce the Path-Adapted Coordinated System (PACS), a novel method for transforming 2D location data from group movement into a path-based coordinate system. Instead of using the immediate heading direction of the centroid at each timepoint, we use the full path of the centroid to transform the coordinates. Here the y-axis denotes movement along the group's path (+ y forward, −y backward) and the x-axis denotes movement to the left or right orthogonal to the group’s forward direction. We apply this transformation to each movement period separately. We use the centroid location over time to define the path of the group, but it should be noted that this method can be used with a pre-determined path, such a road or a racing line. The smoothed path of the group is established by fitting a B-spline to the centroid locations (or route coordinates). Once the smooth path is established, we compute each member’s location along the group path as well as lateral distance from the path. To do this, we find the nearest point on the spline for each participant at every timepoint, as well as the nearest point to the centroid on the spline for each timepoint. The PACS-y coordinate is the distance along the spline from the centroid’s spline location to each participant’s spine location. A positive y value indicates being ahead of the group’s centroid, and negative value indicates being behind the centroid. The PACS-x coordinate is the distance from the participant to the spline, with -x indicating the participant is to the left of the path, and + x indicating the participant is to the right of the path. Figure 1 depicts this process applied to Fig. 1 raw UTM locations of a group of team members and the resulting PACS-transformed locations.

PACS transforming the location data forces the two-dimensional location signal to be relative to the group’s instantaneous location and path heading. This allows the application of other spatial metrics such as entropy. The PACS transformation creates a dynamic "playing field" which allows the extension of common field-related metrics to other path-following sports. Potential applications of this technique include racing sports such as running, cycling, sailing, motorsports, and more. This transformation enables the application of many common metrics in sports analytics and ecology, such as individual’s Spatial Exploration Index (SEI) within the group, the length / width ratio of the group, and the entropy of everyone’s location within the group over time. We also introduce some novel metrics, such as the consistency of individual’s locations within the group across different time periods. These features are described further in Sect. 4.4.2.

**Fig. 2:**
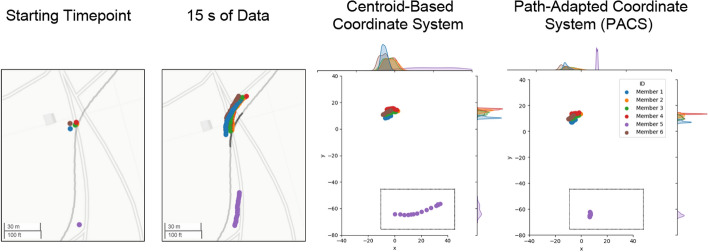
PACS sample scenario: The leftmost figure shows the starting position on a map of all group members. Note that one group member is approximately 60 m behind the rest of the group. The second image from the left shows 30 s of that group’s individual location data while the group encounters a right turn. The third image from the left shows the centroid and heading direction-based transformation. Along the top and right edges, we plot smoothed histogram of everyone’s coordinates. The group member behind the rest of the group shows an inaccurate location within the group yielding an artificially broad histogram across the top edge. In the rightmost figure, the individual's locations are shown in the Path-Adapted Coordinate System where the “swinging” artifact is not present

As noted above, current methods for transforming group movement data rely on the centroid and the centroid’s immediate heading direction, not considering the future and past path of the group/centroid. These transformations cause artifacts in the transformed data, which may in turn cause features extracted from the transformed data to be inaccurate. As an example, consider a case where a group member is located behind the rest of the group (see Fig. 2). When the group begins to round a turn, the centroid direction starts to change before this member has reached the turn, thus resulting in an artificial, and drastic “swing” in that member’s transformed location. The size of this swinging effect is directly related to how far away the individual is from the rest of the group. This artificial motion in the transformed data will affect features such as the Spatial Exploration Index, the length to width ratio, the within-group entropy of the individual as well as the consistency of their group placement across movement periods. Such artifacts would not arise if the group stood abreast such that that they are always facing the group’s heading direction, however this is hardly the case in real-world movement scenarios. We developed PACS to mitigate these artifacts. When directly comparing initial centroid-based transformation and PACS (see Fig. 2), Fig. 2 "swinging” artifacts arise from a group member lagging behind the centroid at a turn. Importantly, the average intra-group location entropy from the group during these 30 s of data is ~ 30% higher in the centroid-based coordinate system.

### Feature details

We employ a comprehensive set of features to characterize time-series movement data, encompassing both individual and group behavior (see Table 1). Individual-level features can be generalized to group-level features through averaging. Feature values over time can be used to calculate associated entropy values from normalized histograms. Furthermore, examining feature consistency across different movement periods offers valuable insights into the group's behavior over time. Here, we describe each feature that was applied to the dataset. A full list of features, descriptions, and feature categories is found in Table 1.

**Table 1:** Group dynamics feature sets

Feature type	Feature	Brief description
Velocity based	Max, mean, and variance of velocity magnitude	Average, maximum, and variance of individual’s velocities during movement
	Max & mean acceleration magnitude	Average and maximum acceleration of individuals during movement
	Velocity difference between team members *	Difference between individual’s velocities within the group
Spatiotemporal	Stretch index*	Average distance of all individuals to the group’s centroid (Folgado et al., 2018)
	Convex hull*—Max, min, mean, var	Convex hull surface area size during movement periods (Moura et al., 2013)
	Voronoi spaces*—Mean, var	Voronoi space that individuals occupy within the convex hull during movement (Rein et al., 2016)
	Length/width ratio*	Length is the Y range of the PACS coordinates and width is the X range
	Spatial exploration index	Quantifies the distance the individual moves from their PACS position centroid during movement (Coutinho et al., 2018; Gonçalves et al., 2017a, 2017b)
Clustering	Time as outliers	The amount of time anyone is an outlier (separated from group) during movement
	Number of outliers	The number of individuals that become outliers at any point during a movement period
	Cluster consistency	Quantifying how consistent the identified cluster members are during a movement period
	Membership confidence (HDBSCAN)	The membership confidence, which relates to the density of the cluster and an individual’s distance to that cluster
	Nearest neighbor*	The nearest neighbor for all individuals. Calculated in Euclidean distance as well as in the PACS X-direction and Y-direction
Leadership	Directional correlation time delay	Used as a leadership score. The time that it takes for an individual’s movement direction to influence a neighbor’s movement direction (Nagy et al., 2010). A positive score means leading while a negative score means following
	Highly Correlated Segments (HCS)	The ratio of time that an individual’s direction is highly correlated with their neighbors
	Leadership instability	The instability of the Directional Correlation Time Delay (Leadership Score) graphs over different movement periods
Movement Regularity	Entropy of PACS locations	The spread of an individual’s PACS location over a movement period
	Error from Vector auto-regression model	The error from a model that predicts future movement from past movement patterns
	Error from Vector auto-regression model with exogenous variables	The error from a model that predicts future movement from past movement patterns, with other individual’s movement in the model
	Entropy of kinematic variables	The spread of kinematic variables over time during movement (26, 52–54)
	Distribution regularity across movement periods	Comparing the distribution on an individual’s PACS locations during movement

^*^Indicates that the feature results in a time-series and has an associated entropy. These features are averaged over the entire event

#### Velocity-based features

Velocity-based features include the average velocity for a participant during each movement period and the variance in velocity magnitude across group members at each timepoint. Acceleration is an important feature that can be very useful in characterizing different movements and even physical load. However, GPS data lack the accuracy required to characterize acceleration precisely. Some soccer studies couple other modalities with GPS to capture acceleration dynamics (Pons et al., 2021) such as video analysis (Cuevas et al., 2020) or a radio-frequency-based local position measurement (LPM) system (Frencken et al., 2010). Because we are limited to GPS recordings, we only focus on features from the velocity calculation, such as average velocity, maximum velocity, and the variance of velocity across team members at any given timepoint. Features related to velocities across team members at each timepoint are extracted as well, such as the difference between the fastest and slowest team members, as well as the variance of all the participant velocities at each timepoint. These time-series features can be averaged for each participant and each group, and the entropy of each feature, calculated over time for each individual, can also be extracted.

#### Spatiotemporal features

**Fig. 3:**
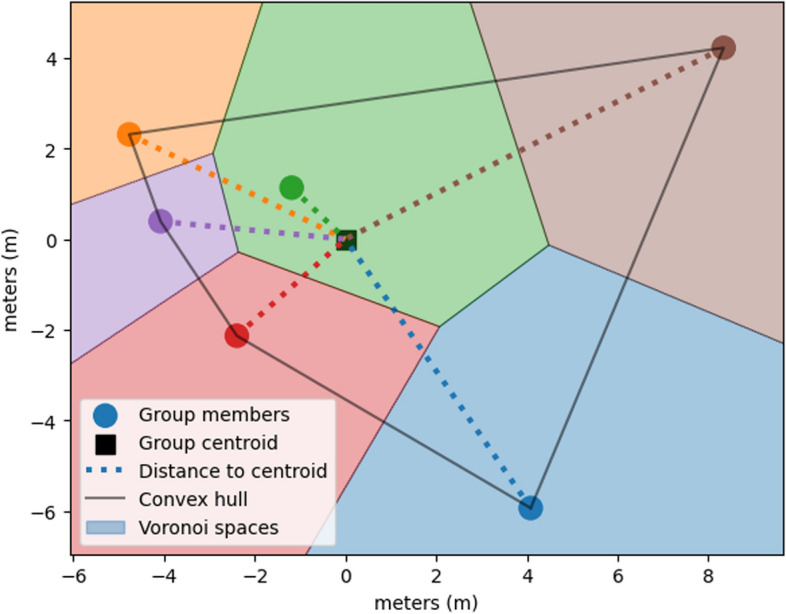
Spatiotemporal features at a single timepoint. Distance of each group member to the centroid, in meters with Voronoi spaces or the "dominated area" of each group member

Spatiotemporal features are important in characterizing tactical behavior in sports (Goes et al., 2021) as well as group dynamics in ecology (Seidel et al., 2018). Common spatiotemporal features in soccer include group distancing metrics such as the team spread and the convex hull area (Moura et al., 2013), which measures the surface area of the smallest convex shape that encloses all group member locations. Another common feature is the Stretch Index, which measures the team spread by finding the distance of each Soldier to the team’s center (centroid) (Folgado et al., 2014). Voronoi diagrams partition a space into areas that surround a set of points; these areas can be used in sports analysis to separate the field into player-dominated areas (Rein et al., 2016). Many group movement activities, such as team members completing a loaded ruck, do not involve a strict "field" boundary. Therefore, to apply the Voronoi space analysis, we combine it with the convex hull boundary to create an area that each participant "dominates" within the group’s covered area at each point in time. This can provide information not only on how much distance is between team members, but also how that space is spread out between each member. If the team members are spaced out evenly in the convex hull, they are expected to have Voronoi spaces of similar size. However, if there is uneven spread within the group (i.e., one pair is close together and another is far apart), then the variance of "dominated" space within the group should increase. The average size of each participant’s Voronoi space within the convex hull as well as the coefficient of variation in the space over time is used as features. A visual example of centroid distances, a convex hull, and Voronoi spaces from a group at one timepoint are shown in Fig. 3.

From PACS-transformed data, we can obtain features common in sports and ecology that require a "stationary" group and a known heading direction. These include an individual’s Spatial Exploration Index within the group (Coutinho et al., 2018; Gonçalves et al., 2017a, 2017b), the length / width ratio of the group, and the entropy of individual’s locations within the group over time. Gonçalves et al. introduced the Spatial Exploration Index (SEI) in a soccer context as the average distance of each player to their own average location (individual centroid). The SEI of individuals is used in soccer (Coutinho et al., 2018; Gonçalves et al., 2017a, 2017b) and basketball analytics (Arede et al., 2021). We compute SEI using the team members’ PACS locations to characterize their intra-group movement. Specifically, we quantify individual differences in SEI (Ramdas et al., 2015) by computing F-statistics and the Wasserstein distances between pairs of normalized distributions in the PACS-x and PACS-y axes for each individual. These metrics are then averaged over movement periods to obtain individual-level features. The individual-level metrics can also be averaged over the group members to yield a group-wide feature.

#### Clustering features

Clustering is an essential part of sports analysis and movement ecology. Clustering methods and distance thresholds vary depending on the specific application. Here, we employ a density-based clustering method (DBSCAN) (Ester et al., 1996) and a hierarchical density-based clustering method (HDBSCAN) (McInnes & Healy, 2017) for identifying groups of individuals at each point in time. These robust clustering methods are chosen because they are density based, which means inclusion of points in a cluster is dependent on the density of the clusters or the compactness of neighboring data points within a dataset.

These methods require a minimum cluster size and distance threshold to be set. The minimum cluster size decides how many members create a cluster while the distance threshold decides how close two clusters should be before they are merged into one. An outlier is defined as a group member(s) who is above the distance threshold from the nearest group. When the minimum cluster size is set to two, we consider only individual members to be outliers. However, if the minimum cluster size is set to be larger, even groups of two or more can be considered outliers. More than one set of parameters can be applied to extract a collection of clustering features and create a more robust feature set. These parameters should be chosen based on the dataset at hand and more than one set of parameters for a more robust feature set.

From these clustering methods, we can extract group metrics such as how many cluster outliers there are, how long these outliers stay separated from clusters, and how consistent cluster memberships are over time. HDBSCAN provides an additional membership confidence metric, which is based on how distant each of the cluster members are in comparison with the density of the cluster. Individual metrics can also be extracted, such as the amount of time one spends as an outlier and the average cluster membership confidence over time. Spatial features can be further refined by implementing clustering methods to identify sub-groups before extracting the spatial features. We do not provide functions for assessing sub-group dynamics and assume groups are never fully separated; however, this is important to consider in applicable scenarios such as a group that splits up to complete separate tasks.

#### Directional leadership features

The influence of individuals within groups is another important aspect of coordinated team movement that can define and characterize leadership hierarchy and collective decision-making. Nagy et al. (2010), introduced a directional correlation method for finding the time delay of directional influence between pigeons in a flock (Nagy et al., 2010). For pairs of birds, they find the correlation between movement directions for different time lags and select the lag (time delay) where there is maximum correlation. From here, they construct a leader–follower hierarchical network where a more positive time delay suggests that others are influenced by the direction of an individual, and a more negative time delay suggests that the individual is influenced by other members’ movement direction. A value can be set for each pairwise relationship as well, quantifying the directional influence time delay between two individuals. This method is also used by Marcelino et al. (2020), in a soccer setting to find Highly Correlated Segments (HCS) and the ratio of HCS time to non-HCS time between neighboring teammates. For a segment of time to be considered a "highly correlated segment," the maximum directional correlation between the two individuals should be above 0.99. The ratio of HCS time for each pair is the amount of time spent highly correlated divided by the amount of time spent in proximity but without highly correlated direction. HCS provides a metric for characterizing pairwise relationships within the team as well as between teammates and opponents (Marcelino et al., 2020). These metrics can be useful in characterizing relationships between team members and about direction influence within a group’s movement patterns.

**Fig. 4:**
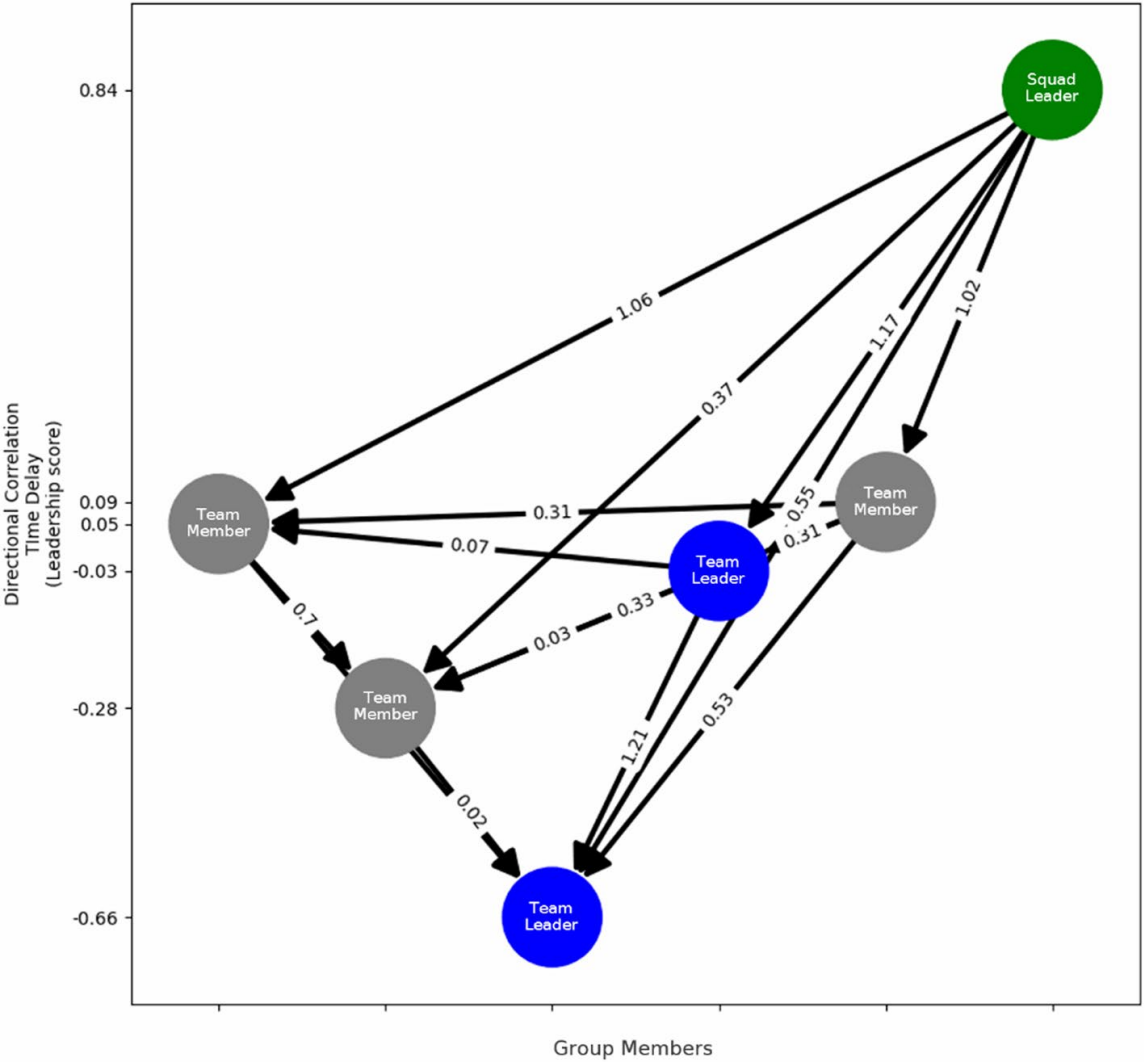
Leadership hierarchy graph from directional correlation time delay analysis. Green nodes indicate a participant is assigned a squad leader position; blue nodes indicate a participant is assigned one of two team leader positions. The location on the y-axis represents the average directional correlation time delay for that individual. The edges represent the direction of influence within a pair of individuals, and the edge value is the magnitude of the directional correlation time delay value for that pair

We implement this directional correlation analysis to rank the group members in terms of intra-team directional influence. From these inferred leadership hierarchies, we derive metrics to determine the effectiveness of the inferred hierarchy. The hierarchy of the movement event is represented as a directed graph, like Nagy et al. where the nodes represent individuals, and the edges represent the correlational time delay pointing from leader to follower. Figure 4 depicts an example leadership hierarchy graph from a group during one movement period. The nodes are organized so that individuals with a higher average correlational time delay (leaders) are at the top and individuals with a lower time delay (followers) are at the bottom. Quality metrics include the ratio of downward facing influence (leader to follower) and the number of loops present in the directed graph (cyclical influence) (Nagy et al., 2010). The consistency of these leadership hierarchy graphs is compared across different movement periods to have a measure of directional leadership stability for each group.

#### Movement regularity features

Stochastic activity can be quantified to provide information about the randomness and complexity of a signal. Entropy is used to quantify the spatial variability of player locations during sports matches as well as the variability of other kinematic variables such as velocity and inter-player distances (Couceiro et al., 2014; Galeano et al., 2021; Rico-González et al., 2020). An individual’s 2D spatial variability is usually characterized by the approximate entropy of their associated normalized histogram. We extract the approximate entropy from the estimated 2D probability density function of each group member’s PACS locations. Bin counts and sizes for entropy calculations can be adjusted to the dataset’s needs but should remain consistent within the complete dataset. This ensures that entropy values across individuals and groups are comparable. For the present human movement data, we used 1 m^2^ bins with a fixed area of around 100 m^2^, similar to the size of a soccer field.

Other applications of Entropy in sports include the analysis of derived kinematic time-series signals, such as using the distance between neighbors (Silva et al., 2014), to get a regularity metric for the positioning relationship of two individuals. Instead of pairwise distances, we use the nearest neighbor in each PACS dimension (X and Y) and find the associated entropy. An entropy value can be extracted from each of the time-series kinematic variables mentioned previously. This is done by creating a histogram of the values present in the time-series from a movement period and then calculating the Approximate Entropy of the distribution. These entropy values can be then assigned to individuals or averaged over the group as a group metric.

Autoregressive modeling has been used to quantify the predictability and randomness of time-series signals (Jain et al., 2016). We apply vector auto-regression models to the 2D movement data to quantify the predictability of some future movement from a window of past movement. We apply a vector autoregressive model (VAR) which includes only one individual’s data and a vector autoregressive model with exogenous variables (VARX) which includes other group member’s movement as exogenous variables to assess the predictability of that individual’s movement with respect to other group movement occurring simultaneously. This predictive modeling is done on a rolling window of 100 samples, where 95 are used for training and 5 are used for prediction. The size of the rolling window to assess predictability upon should be informed by the dataset of interest. The error or residual from these predictive models is used as a metric to assess the predictability of a group member’s movement. The individual metrics are averaged over all group members to generate a group metric.

In the present scenario of a loaded ruck march, team members are expected to be familiar with military doctrine. Specific guidelines are laid out in the Army Techniques Publication (ATP) 3–21.18 titled Foot Marches (Department of the Army, 2022). Some key points in this guideline for foot marches include recommended distances between each participant, the speed at which team members should be moving, and the regularity of break times. ATP 3–21.18 recommends team members should be separated by 2–5 m during the day and march at an approximate speed of 4 km/h (~ 1 m/s). Moreover, this guideline recommends that a 15-min break should be taken after the first 45 min followed by 10-min breaks taken every 50 min following, with variation in break timing kept to a minimum. Consequently, we compute metrics for how closely each of these recommendations is being followed. From team dispersion metrics, we calculate the percentage of time that each participant spends within the recommended distance to a neighbor (between 2 and 5 m apart). We also calculate the time between each break period, taken from the break detection method described previously. The time onset and duration of the breaks should have little variation, so we get the standard deviation from both as two additional metrics. Column length is another important factor during road marches, which quantifies how far the group is spread along the road. We compute this metric from our PACS location data, finding the largest difference in Y locations among the team members.

### Performance prediction

To evaluate the predictive capacity of the discussed metrics, we performed a regression analysis using the STX scores as the dependent variable and group-level metrics from the ruck event as predictors. Given the limited dataset size (*n* = 16), we employed a leave-one-out cross-validation method to estimate model performance. Feature selection was executed using the TRexSelector R package (version 0.0.1) (Machkour et al., 2022). The T-Rex selector is unique in its utilization of dummy variables to regulate the false discovery rate (FDR), thereby ensuring that the expected ratio of falsely identified variables among all selected variables complies with user-defined specifications. This approach inherently mitigates spurious correlations by comparing the significance of real variables against randomly generated dummy variables. This control is maintained while maximizing the selection of variables to bolster the true positive rate (TPR), akin to statistical power (Machkour et al., 2022). We evaluate the performance of this state-of-the-art feature selection method with a standard selection method, least absolute shrinkage, and selection operator (LASSO) regression using a standard stability metric, the Jaccard Stability Index (Khaire & Dhanalakshmi, 2022). This compares the similarity between selected feature sets in each of the leave-one-out iterations.

To validate the linear regression model, we evaluated normality, linearity, independence of residuals, and homoscedasticity. Residual normality was confirmed via a Shapiro–Wilk test (*p* = 0.53) and a near-zero mean residual value (2.44e−15). Linearity was assessed through the observed relationships between predictors and the outcome, which were approximately linear. Residual independence was confirmed with the Ljung–Box test (*p* = 0.087), and the Goldfeld–Quandt test indicated homoscedasticity (*p* = 0.59). Together, these results confirm that linear regression is appropriate for our dataset, aligning with our aim of interpretable and generalizable predictor–outcome relationships.

## Results

### Regression analysis

The group-level metrics derived from the ruck event were leveraged to characterize and predict future STX scores. Two types of regression models were constructed: explanatory and predictive. Explanatory modeling includes all datapoints while predictive modeling leaves out some data in order to assess predictability. Due to the limited number of observations, a leave-one-out approach was adopted to validate the predictive power of the features, allowing for maximal training sample utilization while retaining an untouched sample for evaluation. Within each leave-one-out iteration, a linear regression model was fitted from the scikit-learn library (version 1.4.0) using features selected by the T-Rex selector. We set the false detection rate (FDR) to 0.05 (5%), which is a common threshold as it limits the number of false discoveries while maximizing the discovery of true effects. Subsequently, the performance of the fitted model was assessed using the left-out datum. We compare the performance of the T-Rex selector with a LASSO regression by assessing the similarity of the selected feature sets from each leave-one-out iteration, using Jaccard’s Stability Index (Real & Vargas, 1996). A value close to 0 indicates dissimilar feature sets suggesting instability in the feature selection method. A value close to 1 indicates similar feature sets suggesting stability in the selection method. Our results show the T-Rex selection method achieves a 0.88 while the LASSO selection method achieves a 0.38. This quantifies how much more stable the T-Rex selection method is compared with LASSO, which is a primary reason we selected the T-Rex selection method over alternative options.

### Explanatory model

**Fig. 5:**
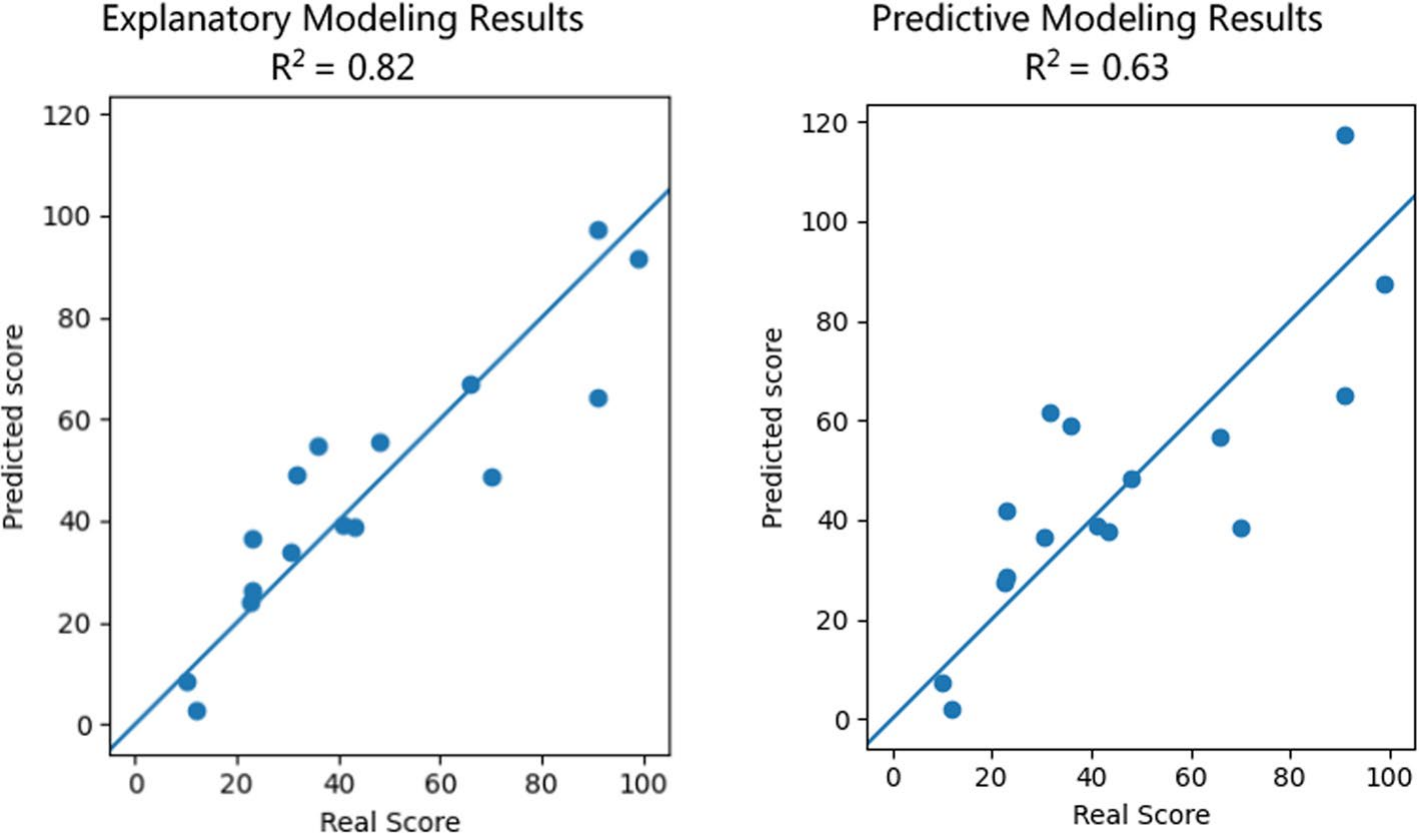
Explanatory and predictive modeling results. The y-axis represents the predicted score while the x-axis represents the real score. Each data point represents one group. The explanatory results (left) show the training results from including all datapoints (groups) in a regression model. The predictive results (right) show the performance of the leave-one-out iterations

The objective of the explanatory modeling phase was to identify key features from the initial 69 independent variables. The results, shown on the left side of Fig. 5, display a plot of the fitted model's output scores (y-axis) against the actual scores (x-axis).

The T-Rex feature selection method selected five of the 69 features. These include the "*reasonable pace ratio*," "*velocity difference entropy*," "*velocity variance*," "*VAR error*," and "*leadership inconsistency*."

The *"reasonable pace ratio"* is the ratio of time spent moving at a reasonable pace, defined as below 1.7 m/s, which is classified as a "fast pace" for humans walking outdoors (Murtagh et al., 2021). This variable has a negative regression coefficient, indicating that groups who move quickly for a significant portion of the ruck tend to perform worse in the STX task compared to those who maintain a more reasonable pace. Military doctrine defines a reasonable pace for a dismounted foot march as approximately 1.3 m/s, a speed that accounts for factors such as terrain, weather, and the load carry (Department of the Army, 2022). Although a faster pace might seem advantageous for covering ground quickly, it often leads to increased physical strain, negatively impacting a unit’s operational performance. Maintaining a more deliberate pace allows Soldiers to conserve energy and sustain group cohesion.

Using these five selected features, we assessed the model's performance based on its fit to the training data, yielding an R-squared value of 0.82, indicating robust explanatory power. The regression results are shown on the left panel of Fig. 4.

The *"velocity difference entropy"* measures the approximate entropy of the difference between the highest and lowest velocities of team members at each time point. This variable has a positive regression coefficient, suggesting that groups with a dynamic range of member speeds perform better in the STX task. This relationship may reflect military doctrine, wherein Soldiers are taught to vary individual and unit spacing based on situational factors such as threat levels, terrain, and visibility (Department of the Army, 2024). These dynamic adjustments could enhance situational awareness, communication, and decision-making under operational conditions, contributing to better performance in the STX task.

Similarly, the *"velocity variance"* is the average variance of team members' velocities over time. This variable also has a positive regression coefficient, indicating that groups perform better when members move at varying speeds during the ruck. In operational scenarios, uniform velocities among group members may increase vulnerability to attack by making their movement more predictable and reducing adaptability to threats or environmental changes. Conversely, varying speeds allow members to dynamically adjust based on their position or team role. These variations in velocity may enhance situational awareness and communication by enabling individuals to respond to emerging threats or obstacles more effectively. From a cognitive perspective, velocity variance reflects the group’s ability to coordinate decision-making and maintain adaptability under dynamic conditions.

Furthermore, the "*VAR error*" is the average error from applying a vector autoregressive model on each participant’s path, which is a measure of movement predictability. This variable has a positive regression coefficient, suggesting that groups with team members who have less random movement direction perform the STX better. While some variability in velocity is tactically advantageous, it remains essential for the unit to move coherently and in coordination with the group leader. Lower VAR error reflects greater predictability among team members, which facilitates shared situational awareness, communication. Teams with more cohesive and disciplined movement are better able to maintain and respond effectively to dynamic conditions, directly contributing to improved performance in the STX task.

Lastly, "*leadership inconsistency*" is a measure of the change between the directional leadership hierarchy between movement periods. More specifically, it is the average pairwise edit distance between the hierarchy graph representations from each of the movement periods. This variable has a positive regression coefficient, suggesting that groups with a more varying leadership hierarchy over the movement periods perform the STX better. This suggests that flexible leadership, where roles and responsibilities shift as needed, enhances group performance. For instance, while the squad leader typically controls unit movement and pace, a Soldier closer to a point of interest (e.g., broken branches indicating recent activity) might temporarily assume leadership to guide the group’s actions. This adaptability reflects a high level of discipline, trust, and distributed decision-making within the unit. By allowing leadership roles to shift based on situational demands, the group can respond more effectively to dynamic conditions, demonstrating cognitive flexibility that directly contributes to mission success.

In summary, a high-performing group strikes a balance between maintaining a reasonable pace and exhibiting dynamic speed differences among members. They also demonstrate variability in speed and leadership hierarchy, enabling movements that are both predictable and adaptable to changing conditions. These findings underscore the importance of adaptability, predictability, and cohesion in STX performance, reflecting critical cognitive processes such as decision-making, situational awareness, and coordination.

### Predictive model

Of the five selected features, four were consistently chosen in every leave-one-out iteration, with one selected in 9 out of 16 iterations. These predictive regression models yielded an R^2^ value of 0.63, denoting their moderate effectiveness in accounting for variance in the outcome. The results from this predictive model are shown on the right side of Fig. 5, where the y-axis represents the predicted score and the x-axis represents the real STX score. Interestingly, the feature exhibiting variability in selection frequency was "*leadership inconsistency*," suggesting its relatively lower importance in predicting group performance compared to the other selected features.

The PACS transformation was applied to enhance spatial feature extraction by incorporating trajectory and heading information for group movement analysis. To evaluate its impact, we assessed the model's performance with and without features derived from the PACS transformation. The explanatory model yielded an R^2^ value of 0.82 with the PACS-derived feature included, indicating robust explanatory power. When features derived from the PACS transformation were excluded, the feature set remained largely unchanged, except for the absence of “Vector-Autoregression error (VAR error),” which relied on the transformation. Notably, “leadership inconsistency” was selected more frequently (10 of 14 iterations vs. 7 of 14 iterations when “VAR error” was included). The explanatory model *R*^2^ decreased slightly to 0.81, and the predictive model *R*^2^ dropped from 0.63 to 0.61.

These results suggest that while the PACS transformation contributes only a modest improvement to model performance, it represents an important step in the feature extraction process for spatially complex data. Its utility is likely to be particularly relevant in future group movement studies involving dynamic or unconstrained environments.

These results suggest that the extracted features can explain and predict the future performance of groups based on their collective movement in an unconstrained space. Future confirmatory research on a larger sample would help validate these findings. However, the present study’s results demonstrate the validity of our features and their predictive value for inferring future performance.

## Discussion

The aim of this study was to develop a comprehensive toolkit for understanding and characterizing group dynamics using location-based metrics during goal-directed activities, particularly focusing on scenarios involving groups of individuals moving in a relatively unbounded environment. By adapting methods from sports analytics and movement ecology, we sought to identify key features and metrics to predict group-level performance on a force-on-force battle drill, contributing to the broader field of location-based analytics and cognitive science. There are four primary outcomes of this work that contribute to extant research and modeling of group dynamics.

First, we outlined a systematic approach for identifying periods of movement and rest within-group data, enabling us to extract meaningful movement-related features while excluding breaks or rest periods. This process reflects cognitive processes such as shared situational awareness, where the identification and coordination of movement and rest periods support group cohesion and energy management. In continuing work, it may be valuable to assess group dynamics and cohesion during rests; for example, periods of time when individuals will cluster into smaller social groups while eating or drinking. These emergent behaviors likely reflect implicit communication, social bonding, and sub-group dynamics, offering additional insights into cognitive and social processes within the larger team. We also employed clustering analysis, leveraging hierarchical density-based clustering (HDBSCAN), to reveal group separations and facilitate the identification of outliers and cluster memberships. These metrics provide insights into the heterogeneity, cohesion, and organization of the group, informing how individual roles and responsibilities within the group emerge and adapt based on collective needs, a hallmark of effective cognitive coordination.

Second, we introduced the Path-Adapted Coordinate System (PACS) to provide a novel framework for transforming GPS data into a coordinate system aligned with the group’s path. This process facilitated extraction of common sports analytics metrics and analysis of group dynamics in relation to the group’s overall movement trajectory. By aligning coordinates with the direction of group movement, PACS enables the analysis of group behavior in relation to the terrain and mission objectives, enhancing the interpretability and utility of the derived metrics. This approach highlights how external factors, such as terrain and objectives, shape internal cognitive processes like decision-making and planning. By aligning group behavior with environmental demands, PACS allows for a deeper understanding of how cognitive and contextual factors interact to influence group performance. This method may be particularly advantageous in situations involving the movement of groups along curvilinear trajectories, including roads, and relating their behavior in a common and relatively interpretable analytic framework. Understanding these relationships can inform strategies to optimize team navigation and adaptability in complex environments, reflecting the cognitive trade-offs between flexibility and structure.

Third, we detailed a comprehensive set of group dynamics features that encompassed velocity-based, spatiotemporal, clustering, directional leadership, and movement regularity metrics. Together, these features offer insights in a range of group dynamics that facilitate a more comprehensive quantification of group behavior over time. These features capture key cognitive aspects of group performance, such as adaptability, cohesion, and shared attention. These metrics shed light on the pacing, consistency, and adaptability of team members during ruck marches, highlighting their importance in determining overall group effectiveness of operational tasks immediately following the completion of the ruck. Spatiotemporal features, including team spread, convex hull area, and Voronoi diagrams adapted to the group's area, offer valuable perspectives on the tactical behavior and spatial organization of the group. Such metrics reflect how cognitive processes like situational awareness, spatial reasoning, and distributed attention are enacted at the group level, shaping tactical readiness and operational success.

Fourth, we used a regression-based analysis and demonstrated the ability to leverage selected collective features to predict groups’ future performance on a task qualitatively different from the ruck march. Identifying key features, such as reasonable pace ratio, velocity dynamics, leadership consistency, and movement predictability, highlighted variables that contribute in a generalizable manner to predicting group effectiveness across tasks and contexts. This result underscores the cognitive consistency of certain group behaviors, where adaptability and cohesion persist across varying operational demands. These findings align with theories of Shared Mental Models and Interactive Team Cognition, suggesting that effective teams develop and maintain shared understanding that supports performance across diverse scenarios.

## Conclusion

Overall, we presented a novel and extensible toolkit for analyzing group movement dynamics via GPS data, offering significant contributions to sports analytics, ecology, and related fields. The comprehensive feature sets calculated by this toolkit enable the extraction of meaningful insights from complex and dynamic human behavior, including relatively holistic understandings of group behavior. Importantly, we also demonstrate the predictive capacity of these features, underscoring their potential utility in forecasting group performance across a wide range of contexts, tasks, and goals. We hope this toolkit lays a helpful foundation for future research endeavors aimed at unraveling the complexities of collective human behavior and cognition.

## Appendix

Here we define the math behind the preprocessing and feature extraction steps.

We define a group movement dataset from a single movement period as:

$$R_{i} \left( {t_{j} } \right) = \left[ {x_{i} \left( {t_{j} } \right) y_{i} \left( {t_{j} } \right)} \right]^{T} \;{\text{for}}\; i = 1,2, \ldots N_{p } \;{\text{and}}\;j = 1,2, \ldots ,N_{t}$$

where

$$N_{p} = {\text{number}}\;{\text{of}}\;{\text{team}} \;{\text{members}}$$

$$N_{t} = {\text{number}}\; {\text{of}}\;{\text{ samples}}$$

$$t_{j} = n\Delta T\;{\text{for}}\; n = 0,1, \ldots ,N_{t} - 1$$

$$\Delta T = {\text{ sampling}}\;{\text{period}}$$

### Smoothing and interpolation

To deal with erroneous and missing values that are inherent to GPS data, we find the first difference in each dimension from an individual’s route ($R_{i}$). We then discard values where the first difference is greater than the 0.99 quantile.

First difference

$${\text{d}}x_{i} \left( {t_{j} } \right) = \left| { x_{i} \left( {t_{j} } \right) - x_{i} \left( {t_{j} - 1} \right)} \right|$$

$${\text{d}}y_{i} \left( {t_{j} } \right) = \left| { y_{i} \left( {t_{j} } \right) - y_{i} \left( {t_{j} - 1} \right)} \right|$$

Dropping points where first difference above 0.99 quartile

$$\hat{x}_{i} \left( {t_{j} } \right) = \left\{ { \begin{array}{*{20}c} {x_{i} \left( {t_{j} } \right),} & {{\text{d}}x_{i} \left( {t_{j} } \right) < {\text{percentile}}\left( {{\text{d}}x_{i} \left( t \right),99} \right)} \\ {NaN,} & {{\text{d}}x_{i} \left( {t_{j} } \right) \ge {\text{percentile}}\left( {{\text{d}}x_{i} \left( t \right),99} \right)} \\ \end{array} } \right.$$

$$\hat{y}_{i} \left( {t_{j} } \right) = \left\{ { \begin{array}{*{20}c} {y_{i} \left( {t_{j} } \right),} & {{\text{d}}y_{i} \left( {t_{j} } \right) < {\text{percentile}}\left( {{\text{d}}y_{i} \left( t \right),99} \right)} \\ {NaN,} & {{\text{d}}y_{i} \left( {t_{j} } \right) \ge {\text{percentile}}\left( {{\text{d}}y_{i} \left( t \right),99} \right)} \\ \end{array} } \right.$$

$${\text{for}}\;j = 1,2, \ldots ,N_{t} \;{\text{and}}\;i = 1,2, \ldots N_{p}$$

To interpolate the missing and discarded timepoints, we employ a linear interpolation technique.

$$\hat{x}_{i} \left( {t_{j} } \right) = \frac{{x_{i} \left( {t_{j} - 1} \right) + x_{i} \left( {t_{j} + 1} \right)}}{2} \;{\text{where}}\; x_{i} \left( {t_{j} } \right) = {\text{NaN}}$$

$$\hat{y}_{i} \left( {t_{j} } \right) = \frac{{y_{i} \left( {t_{j} - 1} \right) + y_{i} \left( {t_{j} + 1} \right)}}{2}\;{\text{ where}}\; y_{i} \left( {t_{j} } \right) = {\text{NaN}}$$

$${\text{for}}\;j = 1,2, \ldots ,N_{t} \;{\text{and}}\;i = 1,2, \ldots N_{P}$$

### Velocity features

To obtain velocity features, we apply a moving average filter to smooth the datapoints further before obtaining the velocity magnitude via Euclidean combination of first differences.

$$\hat{x}_{i} \left( {t_{j} } \right) = \frac{{\mathop \sum \nolimits_{k = j}^{j + w} x_{i} \left( {t_{k} } \right)}}{w}$$

$$\hat{y}_{i} \left( {t_{j} } \right) = \frac{{\mathop \sum \nolimits_{k = j}^{j + w} y_{i} \left( {t_{k} } \right)}}{w}$$

$$w = {\text{window}}\;{\text{length}}$$

$${\text{for}}\;j = 1,2, \ldots ,N_{t} - w$$

Euclidean combination from MA-smoothed first differences

$${\text{d}}\hat{x}_{i} \left( {t_{j} } \right) = \left| {\hat{x}_{i} \left( {t_{j} } \right) - \hat{x}_{i} \left( {t_{j} - 1} \right)} \right|$$

$${\text{d}}\hat{y}_{i} \left( {t_{j} } \right) = \left| {\hat{y}_{i} \left( {t_{j} } \right) - \hat{y}_{i} \left( {t_{j} - 1} \right)} \right|$$

$$V_{i} \left( {t_{j} } \right) = \sqrt {{\text{d}}\hat{x}_{i} \left( {t_{j} } \right)^{2} + {\text{d}}\hat{y}_{i} \left( {t_{j} } \right)^{2} }$$

$${\text{for}}\;j = 1,2, \ldots ,N_{t} \;{\text{and}}\;i = 1,2, \ldots N_{p}$$

Variance of velocity for an individual over the movement period

$${\text{velVar}}_{i} = \sqrt {\frac{{\mathop \sum \nolimits_{j = 1}^{{N_{t} }} \left( {V_{i} \left( {t_{j} } \right) - \overline{{V_{i} \left( t \right)}} } \right)^{2} }}{{N_{t} - 1}}} \;{\text{for}}\;i = 1,2, \ldots N_{p}$$

Velocity variance within squad at each timepoint

$${\text{groupVelVar}}\left( {t_{j} } \right) = \sqrt {\frac{{\mathop \sum \nolimits_{i = 1}^{{N_{p} }} \left( {V_{i} \left( {t_{j} } \right) - \overline{{V\left( {t_{j} } \right)}} } \right)^{2} }}{{N_{p} - 1}} } \;{\text{ for}}\;j = 1,2, \ldots N_{t}$$

Velocity difference within squad

$${\text{velDiff}}\left( {t_{i} } \right) = \mathop {\max }\limits_{{i \in 1,2, \ldots N_{p} }} (V_{i} \left( {t_{j} } \right)) - \mathop {\min }\limits_{{i \in 1,2, \ldots N_{p} }} (V_{i} \left( {t_{j} } \right)) for j = 1,2, \ldots N_{t}$$

### Spatiotemporal features

Centroid over time

$${\text{centroid}}\left( {t_{j} } \right) = \left[ {\frac{{\mathop \sum \nolimits_{i = 1}^{{N_{p} }} x_{i} \left( {t_{j} } \right)}}{{N_{p} }} \frac{{\mathop \sum \nolimits_{i = 1}^{{N_{p} }} y_{i} \left( {t_{j} } \right)}}{{N_{p} }}} \right]^{T} \;{\text{for}}\; j = 1,2, \ldots N_{t}$$

## References

[CR1] Andrienko, N., Andrienko, G., Barrett, L., Dostie, M., & Henzi, P. (2013). Space transformation for understanding group movement. *IEEE Transactions on Visualization and Computer Graphics,**19*(12), 2169–2178. 10.1109/TVCG.2013.193 24051783 10.1109/TVCG.2013.193

[CR2] Arede, J., Cumming, S., Johnson, D., & Leite, N. (2021). The effects of maturity matched and un-matched opposition on physical performance and spatial exploration behavior during youth basketball matches. *PLoS ONE,**16*(4), e0249739. 10.1371/journal.pone.0249739 33831106 10.1371/journal.pone.0249739 PMC8031392

[CR3] Bastille-Rousseau, G., Potts, J. R., Yackulic, C. B., Frair, J. L., Ellington, E. H., & Blake, S. (2016). Flexible characterization of animal movement pattern using net squared displacement and a latent state model. *Movement Ecology,**4*, 15. 10.1186/s40462-016-0080-y 27252856 10.1186/s40462-016-0080-y PMC4888472

[CR4] Bloch, A. E., Steckenrider, J. J., Zifchock, R. A., Freisinger, G. M., Bode, V. G., & Elkin-Frankston, S. (2023). Effect of fatigue on movement patterns during a loaded ruck march. *Military Medicine*. 10.1093/milmed/usad086 10.1093/milmed/usad086 37083060

[CR5] Bunnefeld, N., Börger, L., van Moorter, B., Rolandsen, C. M., Dettki, H., Solberg, E. J., & Ericsson, G. (2011). A model-driven approach to quantify migration patterns: Individual, regional and yearly differences. *The Journal of Animal Ecology,**80*(2), 466–476. 10.1111/j.1365-2656.2010.01776.x 21105872 10.1111/j.1365-2656.2010.01776.x

[CR6] Bustos, D., Guedes, J. C., Vaz, M. P., Pombo, E., Fernandes, R. J., Costa, J. T., & Baptista, J. S. (2021). Non-invasive physiological monitoring for physical exertion and fatigue assessment in military personnel: A systematic review. *International Journal of Environmental Research and Public Health,**18*(16), 16. 10.3390/ijerph18168815 10.3390/ijerph18168815 PMC8393315 34444564

[CR7] Calenge, C., Dray, S., & Royer-Carenzi, M. (2009). The concept of animals’ trajectories from a data analysis perspective. *Ecological Informatics,**4*(1), 34–41. 10.1016/j.ecoinf.2008.10.002

[CR8] Cannon-Bowers, J. A., Salas, E., & Converse, S. (1993). Shared mental models in expert team decision making. *Individual and Group Decision Making: Current Issues,**221*, 221–246.

[CR9] Cheng, K. C., Miller, E. L., Hughes, M. C., & Aeron, S. (2020). On matched filtering for statistical change point detection. *IEEE Open Journal of Signal Processing,**1*, 159–176. 10.1109/OJSP.2020.3035070

[CR10] Cooke, N. J., Cohen, M. C., Fazio, W. C., Inderberg, L. H., Johnson, C. J., Lematta, G. J., Peel, M., & Teo, A. (2023). From teams to teamness: Future directions in the science of team cognition. *Human Factors,**66*(6), 1669. 10.1177/00187208231162449 36946439 10.1177/00187208231162449 PMC11044519

[CR11] Cooke, N. J., Gorman, J. C., Myers, C. W., & Duran, J. L. (2013). Interactive team cognition. *Cognitive Science,**37*(2), 255–285. 23167661 10.1111/cogs.12009

[CR12] Couceiro, M., Clemente, F., Martins, F., & Machado, J. (2014). Dynamical stability and predictability of football players: The study of one match. *Entropy,**16*(2), 645–674. 10.3390/e16020645

[CR13] Coutinho, D., Santos, S., Gonçalves, B., Travassos, B., Wong, D. P., Schöllhorn, W., & Sampaio, J. (2018). The effects of an enrichment training program for youth football attackers. *PLoS ONE,**13*(6), e0199008. 10.1371/journal.pone.0199008 29897985 10.1371/journal.pone.0199008 PMC5999098

[CR14] Cuevas, C., Quilón, D., & García, N. (2020). Techniques and applications for soccer video analysis: A survey. *Multimedia Tools and Applications,**79*(39–40), 29685–29721. 10.1007/s11042-020-09409-0

[CR15] Cushman, S. A., & Huettmann, F. (Eds.). (2010). *Spatial complexity, informatics, and wildlife conservation*. Springer. 10.1007/978-4-431-87771-4

[CR16] Department of the Army. (2016). *INFANTRY PLATOON and SQUAD ATP 3–21. 8*. Headquarters, Department of the Army.

[CR17] Department of the Army. (2022). *Foot Marches (ATP 3–21.18)* (ATP 3–21.18). Headquarters, Department of the Army. https://armypubs.army.mil/epubs/DR_pubs/DR_a/ARN35163-ATP_3-21.18-000-WEB-1.pdf

[CR18] Department of the Army. (2024). *Infantry Platoon and Squad (ATP 3–21.8)* (ATP 3–21.8). Headquarters, Department of the Army. https://armypubs.army.mil/ProductMaps/PubForm/Details.aspx?PUB_ID=1028215

[CR19] Duffield, R., Reid, M., Baker, J., & Spratford, W. (2010). Accuracy and reliability of GPS devices for measurement of movement patterns in confined spaces for court-based sports. *Journal of Science and Medicine in Sport,**13*(5), 523–525. 10.1016/j.jsams.2009.07.003 19853507 10.1016/j.jsams.2009.07.003

[CR20] Early, J. J., & Sykulski, A. M. (2020). Smoothing and interpolating noisy GPS data with smoothing splines. *Journal of Atmospheric and Oceanic Technology,**37*(3), 449–465. 10.1175/JTECH-D-19-0087.1

[CR21] Ester, M., Kriegel, H.-P., Sander, J., & Xu, X. (1996). A density-based algorithm for discovering clusters in large spatial databases with noise. In *Proceedings of the second international conference on knowledge discovery and data mining* (pp. 226–231).

[CR22] Farine, D. R., & Whitehead, H. (2015). Constructing, conducting and interpreting animal social network analysis. *The Journal of Animal Ecology,**84*(5), 1144–1163. 10.1111/1365-2656.12418 26172345 10.1111/1365-2656.12418 PMC4973823

[CR23] Folgado, H., Gonçalves, B., & Sampaio, J. (2018). Positional synchronization affects physical and physiological responses to preseason in professional football (soccer). *Research in Sports Medicine,**26*(1), 51–63. 10.1080/15438627.2017.1393754 29058465 10.1080/15438627.2017.1393754

[CR24] Folgado, H., Lemmink, K. A. P. M., Frencken, W., & Sampaio, J. (2014). Length, width and centroid distance as measures of teams tactical performance in youth football. *European Journal of Sport Science,**14*(Suppl 1), S487-492. 10.1080/17461391.2012.730060 24444244 10.1080/17461391.2012.730060

[CR25] Frencken, W., Lemmink, K., & Delleman, N. J. (2010). Soccer-specific accuracy and validity of the local position measurement (LPM) system. *Journal of Science and Medicine in Sport,**13*(6), 641–645. 10.1016/j.jsams.2010.04.003 20594910 10.1016/j.jsams.2010.04.003

[CR26] Frencken, W., Lemmink, K., Delleman, N., & Visscher, C. (2011). Oscillations of centroid position and surface area of soccer teams in small-sided games. *European Journal of Sport Science,**11*(4), 215–223. 10.1080/17461391.2010.499967

[CR27] Galeano, J., Gomez, M. -Á., Rivas, F., & Buldú, J. M. (2021). Entropy of badminton strike positions. *Entropy,**23*(7), 799. 10.3390/e23070799 34201859 10.3390/e23070799 PMC8304171

[CR28] Gevers, J. M. P., Li, J., Rutte, C. G., & van Eerde, W. (2020). How dynamics in perceptual shared cognition and team potency predict team performance. *Journal of Occupational and Organizational Psychology,**93*(1), 134–157. 10.1111/joop.12287

[CR29] Giles, G. E., Navarro, E., Elkin-Frankston, S., Brunyé, T. T., Elmore, W. R., Seay, J. F., McKenzie, K. L., O’Fallon, K. S., Brown, S. A., Parham, J. L., Garlie, T. N., DeSimone, L., Villa, J. D., Choi-Rokas, H. E., Mitchell, K. B., Racicot, K., Soares, J. W., Caruso, C., Anderson, D., & Eddy, M. D. (2023). Characterizing relationships among the cognitive, physical, social-emotional, and health-related traits of military personnel. *Military Medicine*. 10.1093/milmed/usad002 36705463 10.1093/milmed/usad002

[CR30] Goes, F. R., Meerhoff, L. A., Bueno, M. J. O., Rodrigues, D. M., Moura, F. A., Brink, M. S., Elferink-Gemser, M. T., Knobbe, A. J., Cunha, S. A., Torres, R. S., & Lemmink, K. A. P. M. (2021). Unlocking the potential of big data to support tactical performance analysis in professional soccer: A systematic review. *European Journal of Sport Science,**21*(4), 481–496. 10.1080/17461391.2020.1747552 32297547 10.1080/17461391.2020.1747552

[CR31] Gonçalves, B., Coutinho, D., Santos, S., Lago-Penas, C., Jiménez, S., & Sampaio, J. (2017a). Exploring team passing networks and player movement dynamics in youth association football. *PLoS ONE,**12*(1), e0171156. 10.1371/journal.pone.0171156 28141823 10.1371/journal.pone.0171156 PMC5283742

[CR32] Gonçalves, B., Esteves, P., Folgado, H., Ric, A., Torrents, C., & Sampaio, J. (2017b). Effects of pitch area-restrictions on tactical behavior, physical, and physiological performances in soccer large-sided games. *Journal of Strength and Conditioning Research,**31*(9), 2398–2408. 10.1519/JSC.0000000000001700 27806007 10.1519/JSC.0000000000001700

[CR33] Gorman, J. C., Grimm, D. A., Stevens, R. H., Galloway, T., Willemsen-Dunlap, A. M., & Halpin, D. J. (2020). Measuring real-time team cognition during team training. *Human Factors,**62*(5), 825–860. 10.1177/0018720819852791 31211924 10.1177/0018720819852791

[CR34] Graser, A. (2019). MovingPandas: Efficient structures for movement data in python. *Gi_forum,**1*, 54–68. 10.1553/giscience2019_01_s54

[CR35] Gurarie, E., Andrews, R. D., & Laidre, K. L. (2009). A novel method for identifying behavioural changes in animal movement data. *Ecology Letters,**12*(5), 395–408. 10.1111/j.1461-0248.2009.01293.x 19379134 10.1111/j.1461-0248.2009.01293.x

[CR36] Gurarie, E., Bracis, C., Delgado, M., Meckley, T. D., Kojola, I., & Wagner, C. M. (2016). What is the animal doing? Tools for exploring behavioural structure in animal movements. *Journal of Animal Ecology,**85*(1), 69–84. 10.1111/1365-2656.12379 25907267 10.1111/1365-2656.12379

[CR37] Halson, S. L. (2014). Monitoring training load to understand fatigue in athletes. *Sports Medicine,**44*(Suppl 2), 139–147. 10.1007/s40279-014-0253-z 10.1007/s40279-014-0253-z PMC4213373 25200666

[CR38] Hoenner, X., Whiting, S. D., Hindell, M. A., & McMahon, C. R. (2012). Enhancing the use of Argos satellite data for home range and long distance migration studies of marine animals. *PLoS ONE,**7*(7), e40713. 10.1371/journal.pone.0040713 22808241 10.1371/journal.pone.0040713 PMC3395646

[CR39] Hollingshead, A. B. (1998). Retrieval processes in transactive memory systems. *Journal of Personality and Social Psychology,**74*(3), 659.

[CR40] Jain, A., Abbas, B., Farooq, O., & Garg, S. K. (2016). Fatigue detection and estimation using auto-regression analysis in EEG. In *2016 international conference on advances in computing, communications and informatics (ICACCI)* (pp. 1092–1095). 10.1109/ICACCI.2016.7732190

[CR41] Khaire, U. M., & Dhanalakshmi, R. (2022). Stability of feature selection algorithm: A review. *Journal of King Saud University - Computer and Information Sciences,**34*(4), 1060–1073. 10.1016/j.jksuci.2019.06.012

[CR42] Linke, D., Link, D., & Lames, M. (2018). Validation of electronic performance and tracking systems EPTS under field conditions. *PLoS ONE,**13*(7), e0199519. 10.1371/journal.pone.0199519 30036364 10.1371/journal.pone.0199519 PMC6056042

[CR43] Low, B., Coutinho, D., Gonçalves, B., Rein, R., Memmert, D., & Sampaio, J. (2020). A systematic review of collective tactical behaviours in football using positional data. *Sports Medicine,**50*(2), 343–385. 10.1007/s40279-019-01194-7 31571155 10.1007/s40279-019-01194-7

[CR44] Machkour, J., Muma, M., & Palomar, D. P. (2022). *The Terminating-Random Experiments Selector: Fast High-Dimensional Variable Selection with False Discovery Rate Control* (arXiv:2110.06048). arXiv. http://arxiv.org/abs/2110.06048

[CR45] Marcelino, R., Sampaio, J., Amichay, G., Gonçalves, B., Couzin, I. D., & Nagy, M. (2020). Collective movement analysis reveals coordination tactics of team players in football matches. *Chaos, Solitons & Fractals,**138*, 109831. 10.1016/j.chaos.2020.109831

[CR46] McInnes, L., & Healy, J. (2017). Accelerated hierarchical density based clustering. *IEEE International Conference on Data Mining Workshops (ICDMW),**2017*, 33–42. 10.1109/ICDMW.2017.12

[CR47] Moura, F. A., Martins, L. E. B., Anido, R. O., Ruffino, P. R. C., Barros, R. M. L., & Cunha, S. A. (2013). A spectral analysis of team dynamics and tactics in Brazilian football. *Journal of Sports Sciences,**31*(14), 1568–1577. 10.1080/02640414.2013.789920 23631771 10.1080/02640414.2013.789920

[CR48] Murtagh, E. M., Mair, J. L., Aguiar, E., Tudor-Locke, C., & Murphy, M. H. (2021). Outdoor walking speeds of apparently healthy adults: A systematic review and meta-analysis. *Sports Medicine,**51*(1), 125–141. 10.1007/s40279-020-01351-3 33030707 10.1007/s40279-020-01351-3 PMC7806575

[CR49] Nagy, M., Ákos, Z., Biro, D., & Vicsek, T. (2010). Hierarchical group dynamics in pigeon flocks. *Nature,**464*(7290), 7290. 10.1038/nature08891 10.1038/nature08891 20376149

[CR50] Nathan, R., Getz, W. M., Revilla, E., Holyoak, M., Kadmon, R., Saltz, D., & Smouse, P. E. (2008). A movement ecology paradigm for unifying organismal movement research. *Proceedings of the National Academy of Sciences,**105*(49), 19052–19059. 10.1073/pnas.0800375105 10.1073/pnas.0800375105 PMC2614714 19060196

[CR51] Olthof, S. B. H., Frencken, W. G. P., & Lemmink, K. A. P. M. (2018). Match-derived relative pitch area changes the physical and team tactical performance of elite soccer players in small-sided soccer games. *Journal of Sports Sciences,**36*(14), 1557–1563. 10.1080/02640414.2017.1403412 29125029 10.1080/02640414.2017.1403412

[CR52] Pons, E., García-Calvo, T., Cos, F., Resta, R., Blanco, H., López Del Campo, R., Díaz-García, J., & Pulido-González, J. J. (2021). Integrating video tracking and GPS to quantify accelerations and decelerations in elite soccer. *Scientific Reports,**11*(1), 18531. 10.1038/s41598-021-97903-2 34535734 10.1038/s41598-021-97903-2 PMC8448836

[CR53] Ramdas, A., Garcia, N., & Cuturi, M. (2015). *On Wasserstein Two Sample Testing and Related Families of Nonparametric Tests*. 10.48550/ARXIV.1509.02237

[CR54] Ravankar, A., Ravankar, A., Kobayashi, Y., Hoshino, Y., & Peng, C.-C. (2018). Path smoothing techniques in robot navigation: State-of-the-art. *Current and Future Challenges. Sensors,**18*(9), 3170. 10.3390/s18093170 30235894 10.3390/s18093170 PMC6165411

[CR55] Real, R., & Vargas, J. M. (1996). The probabilistic basis of Jaccard’s index of similarity. *Systematic Biology,**45*(3), 380–385. 10.1093/sysbio/45.3.380

[CR56] Rein, R., Raabe, D., Perl, J., & Memmert, D. (2016). Evaluation of changes in space control due to passing behavior in elite soccer using Voronoi-cells. In P. Chung, A. Soltoggio, C. W. Dawson, Q. Meng, & M. Pain (Eds.), *Proceedings of the 10th International Symposium on Computer Science in Sports (ISCSS)* (Vol. 392, pp. 179–183). Springer. 10.1007/978-3-319-24560-7_23

[CR57] Rico-González, M., Pino-Ortega, J., Nakamura, F. Y., Moura, F. A., & Los Arcos, A. (2020). Identification, computational examination, critical assessment and future considerations of distance variables to assess collective tactical behaviour in team invasion sports by positional data: A systematic review. *International Journal of Environmental Research and Public Health,**17*(6), 1952. 10.3390/ijerph17061952 32192000 10.3390/ijerph17061952 PMC7143020

[CR58] Robles-Palazón, F. J., Comfort, P., Ripley, N. J., Herrington, L., Bramah, C., & McMahon, J. J. (2023). Force plate methodologies applied to injury profiling and rehabilitation in sport: A scoping review protocol. *PLoS ONE,**18*(10), e0292487. 10.1371/journal.pone.0292487 37812631 10.1371/journal.pone.0292487 PMC10561863

[CR59] Rossi, A., Pappalardo, L., Cintia, P., Iaia, F. M., Fernàndez, J., & Medina, D. (2018). Effective injury forecasting in soccer with GPS training data and machine learning. *PLoS ONE,**13*(7), e0201264. 10.1371/journal.pone.0201264 30044858 10.1371/journal.pone.0201264 PMC6059460

[CR60] Seidel, D. P., Dougherty, E., Carlson, C., & Getz, W. M. (2018). Ecological metrics and methods for GPS movement data. *International Journal of Geographical Information Science : IJGIS,**32*(11), 2272–2293. 10.1080/13658816.2018.1498097 30631244 10.1080/13658816.2018.1498097 PMC6322554

[CR61] Silva, P., Travassos, B., Vilar, L., Aguiar, P., Davids, K., Araújo, D., & Garganta, J. (2014). Numerical relations and Skill level constrain co-adaptive behaviors of agents in sports teams. *PLoS ONE,**9*(9), e107112. 10.1371/journal.pone.0107112 25191870 10.1371/journal.pone.0107112 PMC4156427

[CR62] Tang, W., & Bennett, D. A. (2010). Agent-based modeling of animal movement: A review. *Geography Compass,**4*(7), 682–700. 10.1111/j.1749-8198.2010.00337.x

[CR63] Thomsen, P. F., Kielgast, J., Iversen, L. L., Wiuf, C., Rasmussen, M., Gilbert, M. T. P., Orlando, L., & Willerslev, E. (2012). Monitoring endangered freshwater biodiversity using environmental DNA. *Molecular Ecology,**21*(11), 2565–2573. 10.1111/j.1365-294X.2011.05418.x 22151771 10.1111/j.1365-294X.2011.05418.x

[CR64] Toader, A. F., & Martin, R. (2023). Bringing the cognitive revolution forward: What can team cognition contribute to our understanding of leadership? *The Leadership Quarterly,**34*(1), 101619. 10.1016/j.leaqua.2022.101619

[CR65] Torres-Ronda, L., Beanland, E., Whitehead, S., Sweeting, A., & Clubb, J. (2022). Tracking systems in team sports: A narrative review of applications of the data and sport specific analysis. *Sports Medicine - Open,**8*(1), 15. 10.1186/s40798-022-00408-z 35076796 10.1186/s40798-022-00408-z PMC8789973

[CR66] Vanrenterghem, J., Nedergaard, N. J., Robinson, M. A., & Drust, B. (2017). Training load monitoring in team sports: A novel framework separating physiological and biomechanical load-adaptation pathways. *Sports Medicine,**47*(11), 2135–2142. 10.1007/s40279-017-0714-2 28283992 10.1007/s40279-017-0714-2

[CR67] Ye, S., Feng, S., Huang, L., & Bian, S. (2020). Recent progress in wearable biosensors: From healthcare monitoring to sports analytics. *Biosensors,**10*(12), 205. 10.3390/bios10120205 33333888 10.3390/bios10120205 PMC7765261

[CR68] Zhang, J., Qu, Q., & Chen, X.-B. (2023). A review on collective behavior modeling and simulation: Building a link between cognitive psychology and physical action. *Applied Intelligence,**53*(21), 25954–25983. 10.1007/s10489-023-04924-7

